# Genome-Wide Identification, Phylogeny and Expression Analysis of the Magnesium Release Gene Family in Wheat (*Triticum aestivum* L.)

**DOI:** 10.3390/cimb47110882

**Published:** 2025-10-23

**Authors:** Yuanxue Chen, Weiwei Zhang, Fengjuan Zhao, Guolan Liu, Deyong Zhao, Jikun Xu, Xin Wang, Xuehui Zong, Jingmin Zhang, Xiaoqing Ji, Jingyi Ma, Shuaipeng Zhao, Jian Li

**Affiliations:** 1 College of Biological and Pharmaceutical Engineering, Shandong University of Aeronautics, Binzhou 256600, China19550979973@163.com (X.J.); 18764395879@163.com (J.M.); 2 Shandong Key Laboratory of Eco-Environmental Science for the Yellow River Delta, Shandong University of Aeronautics, Binzhou 256600, China

**Keywords:** TaMGR, magnesium, transporter, wheat, stress

## Abstract

Magnesium (Mg) release (MGR) proteins play a crucial role in maintaining Mg^2+^ homeostasis in plant cells. However, *MGR* family genes have not yet been explored in crops. This study identified the wheat MGR (TaMGR) family members via BlastP alignment. A total of 15 *MGR* genes were mapped to 12 chromosomes. Cis-element prediction in the promoter region revealed that the ABA-responsive element (ABRE) was 100% conserved among all family members. Collinearity analysis indicates that *MGR* genes in monocot plants may have higher conservation compared to dicot plants. Expression profiling analyses uncovered the expression patterns of *TaMGR* genes across diverse tissues and under various stresses. Our results demonstrated that *TaMGR5D* and *TaMGR5A.2* were significantly induced by both powdery mildew and stripe rust pathogen infections, whereas *TaMGR4A* transcript levels were upregulated in response to drought, heat and their combined stress. These findings indicate that *TaMGRs* may contribute coordinately to the regulation of wheat growth and development as well as adaptive responses to adverse conditions through member-specific expression patterns. This study systematically identified and analyzed the evolution and expression regulation characteristics of *TaMGRs*, providing a theoretical basis for in-depth research on the functional mechanisms of the *TaMGRs* and for improving the Mg use efficiency and stress adaptability of wheat via molecular approaches.

## 1. Introduction

Magnesium (Mg) is an essential nutrient for plants, and its ions are the second most abundant cations in plant cells, only surpassed by potassium ions [1,2]. Mg plays crucial biological roles such as the regulation of enzyme activity [3], chloroplast gene expression [4] and chlorophyll synthesis [5]. Studies have shown that approximately three-quarters of the Mg in plant leaves is involved in the structural formation and functional maintenance of ribosomes, thus affecting protein synthesis [6]. Mg also plays a key role in regulating ion homeostasis. For example, Mg cooperates with potassium to regulate the cation–anion balance and acts as an osmotically active ion to affect cell turgor pressure [7]. In agricultural production, Mg deficiency causes physiological abnormalities, such as leaf chlorosis and necrosis, and reduces crop yields in severe cases [8]. However, excess Mg can also inhibit plant growth and development [9]. In conclusion, Mg plays an irreplaceable role in plants.

As a cation, Mg^2+^ does not diffuse freely across membranes and requires specific transport proteins to enter cells and be further distributed to organelles [10,11]. Due to the increasing attention paid to Mg, research on Mg transporters has recently become increasingly extensive. CorA-type Mg transporters are the most extensively studied and were first identified in *Salmonella typhimurium* [12]. They have also been identified in various eukaryotes. This type of Mg transporter is associated with Mg^2+^ influx and efflux [13]. For instance, the MGT family, a class of plant Mg transporters analogous to the CorA type, was first identified in *Arabidopsis thaliana*. The protein encoded by the *AtMGT1* gene localizes to the plasma membrane and mediates Mg uptake in the roots [14]. Its overexpression enhances the tolerance of plants to Mg deficiency and aluminum toxicity [15]. AtMGT10 (also known as AtMRS2-11) localizes to the chloroplast membrane [16], indicating its association with Mg^2+^ uptake and transport in chloroplasts. AtMGT5 localizes in mitochondria and mediates Mg transport between the cytoplasm and mitochondria [17]. *AtMGT6* plays a critical role in responding to Mg deficiency and functions in concert with *MGT7* to regulate Mg^2+^ transport [18]. *AtMGT7* generates two mRNA splice variants (*AtMGT7a* and *AtMGT7b*) with distinctly different functions; *AtMGT7a* is expressed in all organs, whereas *AtMGT7b* is only expressed in roots and flowers [19]. Rice (*Oryza sativa*) has nine MGT family members. The Mg^2+^ transporter OsMGT1 enhances the plant’s salt tolerance by regulating OsHKT1;5 transport activity [20]. OsMGT3 localizes to chloroplasts, and overexpression of this gene enhances the photosynthetic efficiency, thereby affecting rice growth [21]. Maize (*Zea mays*) has 12 putative MGT family members (designated as ZmMGT1–ZmMGT12), which have not yet been characterized [22]. ZmMGT10 is involved in Mg^2+^ transport and has high homology with AtMGT6. *ZmMGT10* is specifically expressed in plant roots, and its expression increases under an insufficient Mg supply [23]. Previous research has identified two *ZmMGT10* subtypes. *ZmMGT10;1* expression is correlated with the Mg^2+^ concentration in plant roots, while that in the plant has no impact on *ZmMGT10;2* expression [24]. In soybean (*Glycine max*), GmMGT4 and GmMGT5 localize to the plasma membrane of root nodule cortical cells, and they may have functional redundancy in root nodule Mg transport activities [25]. Recent research has found that 39 *MGT* genes are distributed across 17 chromosomes in soybean. Under the stress conditions of Mg deficiency and excess, the expression of *GmMGT2* and *GmMGT29* is increased in the leaves of stress-tolerant soybean genotypes [26].

Another category of plant transporters has recently been defined as the Mg release (MGR) family, as they are likely involved in the process of releasing Mg^2+^ from the cytoplasm to the vacuole or extracellular space [27]. This family belongs to the ancient conserved domain proteins (ACDPs), which are referred to as cyclin M-type divalent metal cation transport mediators (CNNMs) in mammals [28]. Members of this family have a domain of unknown function 21 (DUF21) that spans the membrane three or four times and two cystathionine-β-synthase (CBS) domains in the cytoplasmic region [29]. Nine members (MGR1–MGR9) of the MGR family have been identified in *Arabidopsis thaliana* [27]. Among them, MGR1, MGR2 and MGR3 localize to the tonoplast, and *MGR1* is a key gene for maintaining Mg homeostasis. There is functional differentiation among the remaining members. For example, MGR4–MGR7 localize to the plasma membrane and are mainly expressed in root stele cells [30]. They participate in long-distance Mg^2+^ transport from roots to aerial parts by mediating Mg^2+^ loading into the xylem. Among these, MGR4 and MGR6 play a dominant role in maintaining Mg homeostasis in aerial parts, and functional loss impairs the plant’s tolerance to both high and low Mg stress. MGR8 and MGR9 localize to the inner membrane of chloroplasts, where they regulate Mg^2+^ homeostasis [31]. They play key roles in embryo development, thylakoid formation and photosynthetic complex assembly and are essential proteins for maintaining chloroplast Mg homeostasis throughout the life cycle of *Arabidopsis thaliana*. One study has revealed that MGR8 and MGR9 belong to the CorC-type transporter family; specifically, *MGR9* functions at a lower Mg concentration (0.5 mM), with a wider range of active concentrations than *MGR8* [10]. Additionally, the Mg transport activities of MGR8 and MGR9 are not inhibited by Al^3+^. *MGR8* is highly expressed in leaves, and *MGR9* is mainly upregulated under Mg deficiency or excess conditions. The discovery of this family has filled the gap in the molecular mechanism of Mg transport in plants, providing key insights for understanding Mg homeostasis and adaptation to related stresses in plants. However, this family has not been reported in other plants, especially in crops.

Since its domestication, wheat (*Triticum aestivum* L.) has spread across the globe and become one of the world’s most important food crops [32], and its yield and quality are directly linked to food security. As an indispensable nutrient for wheat growth and development [33], Mg is involved in key physiological processes, such as plant photophosphorylation, photosynthetic carbon assimilation and metabolic regulation, and plays an important role in photosynthate distribution and utilization [34]. Mg transporters are the core carriers for maintaining Mg homeostasis in wheat, facilitating Mg^2+^ accumulation at specific cellular locations. For instance, as the primary intracellular storage compartment for Mg^2+^, vacuoles require the mediation of Mg transporters to enable the dynamic exchange of Mg ions with the cytoplasm, thus maintaining Mg ion homeostasis in the cytoplasm [35]. Most studies on Mg transporters have focused on model plants, such as *Arabidopsis thaliana* and rice, and studies on Mg transporters in wheat remain limited. Researchers have identified multiple *TaMGT* members and found that the expression levels of some of these genes are significantly upregulated under low-Mg stress, indicating that they may be involved in Mg-deficient response [36]. However, the MGR family has not been reported in wheat. Focusing on the MGR family, this study systematically identified the relevant genes in wheat through gene screening, localization and expression pattern analysis. The results of this study provide theoretical support for exploring the biological functions of wheat *MGR* genes and the genetic basis of wheat’s adaptation to Mg-deficient environments and have significant theoretical value for ensuring sustainable wheat production.

## 2. Materials and Methods

### 2.1. Screening and Identification of MGR Family Members in Wheat

In this study, the protein sequence information of the nine known members of the *Arabidopsis thaliana* MGR family was downloaded from the TAIR database (https://www.arabidopsis.org/, accessed on 4 March 2025). A BlastP search (*E*-value < 10^−5^) was performed on the NCBI website (https://www.ncbi.nlm.nih.gov/, accessed on 4 March 2025), where *Triticum aestivum* (taxid: 4565) was selected as the wheat database. Duplicate and erroneous results were removed. The initially obtained results were screened using the InterPro (https://www.ebi.ac.uk/interpro/, accessed on 9 March 2025) [37] and SMART databases (http://smart.embl-heidelberg.de/, accessed on 9 March 2025) [38] with default parameters to identify protein domains containing the DUF21 domain and two CBS domains. Thus, TaMGR family members were identified. The transmembrane domains of TaMGR family members were predicted using the TMHMM Server v2.0 database (https://services.healthtech.dtu.dk/services/TMHMM-2.0/, accessed on 11 March 2025) [39]. The molecular weight and isoelectric point of the proteins was queried and predicted using the Expasy-ProtParam database (https://web.expasy.org/protparam/, accessed on 19 March 2025) [40]. Subcellular localization prediction of TaMGR family members was performed using the DeepLoc-2.1 prediction tool (https://services.healthtech.dtu.dk/services/DeepLoc-2.1/, accessed on 22 March 2025) [41] combined with the Plant-mPLoc online platform (http://www.csbio.sjtu.edu.cn/bioinf/plant-multi/, accessed on 22 March 2025) [42].

### 2.2. Construction of the Phylogenetic Tree and Analysis of Protein Conserved Motifs in MGR Family

The protein sequences of each TaMGR member were downloaded from the NCBI website (https://www.ncbi.nlm.nih.gov/, accessed on 6 March 2025). A phylogenetic tree was constructed with MEGA12 software [43] using TaMGR sequences and the protein sequences of 9 *Arabidopsis thaliana* MGRs, 4 *Oryza sativa* MGRs, 4 *Sorghum bicolor* MGRs, 10 *Medicago truncatula MGRs* and 10 *Solanum lycopersicum* MGRs. The neighbor-joining method (NJ) was used, with the bootstrap value set to 1000 and the remaining parameters set as the default. The iTOL online visualization tool (https://itol.embl.de/, accessed on 24 April 2025) [44] was then used to optimize the visualization of the phylogenetic tree. Multiple sequence alignment of each TaMGR family member was performed using DNAMAN software 6.0, and annotations were performed based on the prediction of transmembrane domains and functional domains. To reveal the protein functions and structures and clarify their evolutionary relationships and biological mechanisms, the MEME Suite database (https://web.mit.edu/meme/current/share/doc/overview.html, accessed on 28 April 2025) [45] was used to identify conserved motifs in the wheat MGR family, with the maximum number of motifs set to 20 and the remaining parameters set as the default. The TVBOT online tool (https://chiplot.online/tvbot.html, accessed on 29 April 2025) [46] was then used to visualize the motifs.

### 2.3. Chromosomal Localization and Gene Structure Analysis

The GFF3 file of the wheat genome data was downloaded from the NCBI database to obtain the chromosomal localization information of the *TaMGR* family, and TBtools-II v2.357 [47] was used to generate and optimize the chromosomal localization map of the *TaMGR* family. Wheat genome annotation information was obtained from the IWGSC V2.1 wheat genome database (assembled in the NCBI database) to clarify the gene structures of members of this family. TBtools software was used to screen the gene annotation information of this family and generate gene structure diagrams.

### 2.4. Gene Duplication Events and Correlation Analysis

To determine the conserved characteristics of gene sequences and arrangement orders between the wheat *MGR* family and other species as well as among different genomic regions within the species and to clarify the evolutionary history of the genome and associations with gene functions, this study selected monocotyledonous (*Sorghum bicolor* and *Oryza sativa*) and dicotyledonous plants (*Medicago truncatula* and *Glycine max*) for interspecific synteny analysis with wheat and intraspecific collinearity analysis within wheat. Wheat genome data (IWGSC_CS_RefSeq_v2.1) were downloaded from the NCBI database, and genome data for *Medicago truncatula* (MedtrA17_4.0), *Oryza sativa* (Oryza_nivara_v1.0), *Sorghum bicolor* (NCBIv3), and *Glycine max* (Glycine_max_v2.1) were retrieved from the Ensemble plants database. The coding sequences (CDSs) of the target genes in wheat were subjected to blastX alignment against those of *Medicago truncatula*, *Oryza sativa, Sorghum bicolor* and *Glycine max*. Gene pairs with the “reciprocal best hit (RBH)” were retained to eliminate false positives from one-way matches. The following filtering criteria were applied to avoid partial matches: *E*-value < 1 × 10^−10^, identity > 50%, and coverage > 70%. Collinearity plots were generated using the MCScanX plugin in TBtools software.

### 2.5. Cis-Element Analysis

The wheat GFF3 file obtained from the NCBI database was used to reveal the regulatory mechanisms of gene expression and identify cis-acting elements in the promoter regions. The GTF/GFF sequence extractor tool in TBtools software was used to extract the 2000 bp DNA sequence upstream of the *TaMGR* gene promoters. The PlantCare online tool (https://bioinformatics.psb.ugent.be/webtools/plantcare/html//, accessed on 6 May 2025) [48] was used to predict cis-acting elements, and the data were visualized using TBtools software [47].

### 2.6. Analysis of Gene Expression Patterns

To determine the expression patterns of the *TaMGR* gene family, transcriptome datasets encompassing different tissues, developmental stages and stress treatments were obtained from the Wheat Expression Database (http://www.wheat-expression.com/download, accessed on 23 May 2025) [49]. Expression levels were quantified as transcripts per million (TPM) and used for normalization (Supplementary Table S2). For each biological replicate, TPM values were averaged, followed by log2 transformation. A heatmap illustrating gene expression profiles was generated using TBtools software [47].

For validation of gene expression, total RNA was extracted from wheat seedlings using the TransZol Up Plus RNA Kit (TransGen Biotech, Beijing, China). cDNA was synthesized with the TransScript^®^ One-Step gDNA Removal and cDNA Synthesis SuperMix (TransGen Biotech), in accordance with the manufacturer’s instructions. Gene expression analysis was conducted by real-time RT–PCR on a LightCycler 96 system (Roche, Basel, Switzerland) with TranStart Top Green qPCR SuperMix (TransGen Biotech). The gene *Ta2291*(*LOC543336*) was used as an internal reference [50], and primers are provided in Supplementary Table S3. Expression levels were normalized using the 2^−ΔΔCt^ method.

## 3. Results

### 3.1. Screening and Identification of MGR Family Members in Wheat

**Figure 1:**
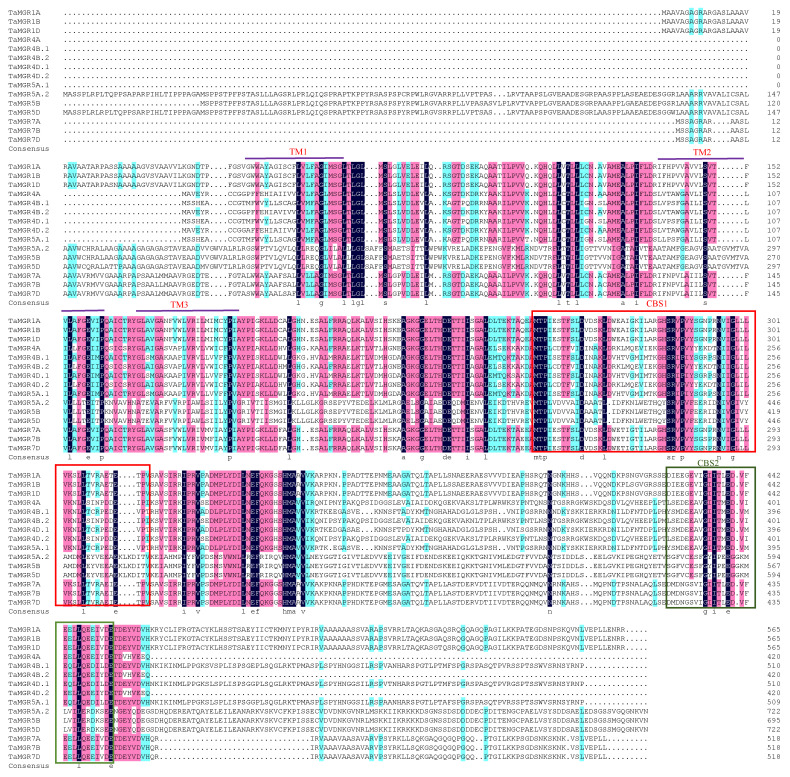
Multiple sequence alignment of the TaMGRs family. TaMGR1A-TaMGR5A.1 and TaMGR7A-TaMGR7D all have three transmembrane domains (TM), with the prediction results labeled in the figure. The transmembrane domains of TaMGR5A.2-TaMGR5D are special and not shown in the figure.

In this study, 15 MGR family members were screened and identified in wheat (Supplementary Table S1). All 15 predicted members contained a DUF21 domain that spanned the membrane three or four times and a pair of CBS domains at the C-terminus. The genes encoding them were localized on different wheat chromosomes, and they were named based on their chromosomal positions. Based on the prediction data, the protein sequence lengths of TaMGR family members ranged from 420 to 723 amino acids. The molecular weight ranged from 46,344.92 to 78,538.18 D, with the number of transmembrane domains varying from 3 to 4. The isoelectric point ranged from 4.70 to 8.13. The protein instability indices were mostly around 40, but those of TaMGR7A, TaMGR7B and TaMGR7D were less than 40, indicating that all 3 proteins were stable. According to the prediction data, TaMGR1A, TaMGR1B, TaMGR1D, TaMGR4A, TaMGR4B.2, TaMGR4D.2, TaMGR7A, TaMGR7B and TaMGR7D were hydrophobic proteins, and the remaining members were hydrophilic proteins. Subcellular localization prediction showed that only three proteins (TaMGR5A.2, TaMGR5B and TaMGR5D) localized to chloroplasts, while the remaining proteins localized to the plasma membrane and vacuolar membrane. The prediction of putative transmembrane domains showed that TaMGR1A–TaMGR5A.1 and TaMGR7A–TaMGR7D all had three transmembrane domains. The prediction results are shown in Figure 1.

### 3.2. Construction of the Phylogenetic Tree and Analysis of Protein Conserved Motifs in the Wheat MGR Family

**Figure 2:**
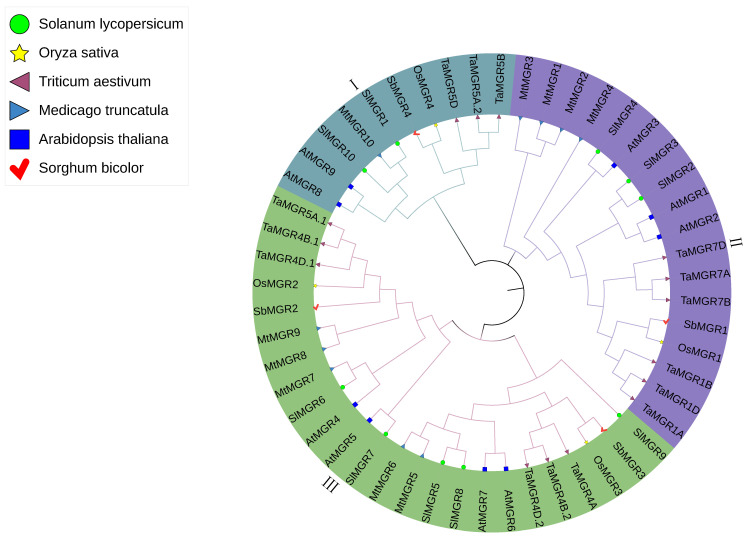
Phylogenetic relationships of MGR family members among *Triticum aestivum*, *Oryza sativa*, *Arabidopsis thaliana*, *Sorghum bicolor*, *Solanum lycopersicum* and *Medicago truncatula*. A neighbor-joining (NJ) phylogenetic tree was constructed using MEGA12 software with 15 TaMGR, 4 OsMGR, 9 AtMGR, 4 SbMGR, 10 SIMMGR and 10 MtMGR proteins.

**Figure 3:**
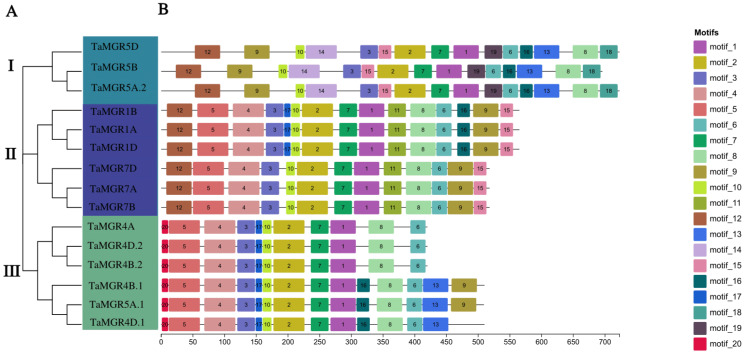
Phylogenetic relationship (**A**) and motif analysis (**B**) of TaMGR family members.

A neighbor-joining phylogenetic tree was constructed to further clarify the phylogenetic relationships between TaMGRs and MGRs from other species, including 15 from wheat, 9 from *Arabidopsis thaliana*, 4 from *Oryza sativa*, 4 from *Sorghum bicolor*, 10 from *Medicago truncatula*, and 10 from *Solanum lycopersicum* (Figure 2). A total of 52 proteins in the phylogenetic tree were divided into 3 clades, with TaMGR members present in each clade. The first clade contained the fewest protein members. The second and third clades had the same number of TaMGR members. A phylogenetic tree was also constructed for the TaMGR family (Figure 3). The 15 MGRs in wheat clustered into three clades (Figure 3A), which was consistent with the results shown in Figure 2. Motif analysis revealed that a total of 20 motifs were predicted (Figure 3B). Among them, three wheat MGR members in Subgroup 1 (TaMGR5A.2, TaMGR5B, and TaMGR5D) lacked motif4 and motif5, whereas all members in Subgroups 2 and 3 possessed these motifs. In contrast, motif14, motif18, and motif19 were uniquely present in the three members of Subgroup 1 and absent in members of Subgroups 2 and 3. Members in Subgroups 1 and 2 all contained motif9 and motif15. In Subgroup 3, only TaMGR4B.1 and TaMGR5A.1 had motif9, while all members in this subgroup lacked motif15. Additionally, motif11 was uniquely present in every member of Subgroup 2. Motif12 was exclusively present in each member of Subgroups 1 and 2, and motif20 was exclusively present in each member of Subgroup 3. In addition, shared motifs, such as motif6, motif7 and motif8, show differences in position both within and between subgroups, which reflects the refined functional division within the protein family and its adaptation to complex biological needs.

### 3.3. Chromosomal Localization and Gene Structure Analysis

**Figure 4:**
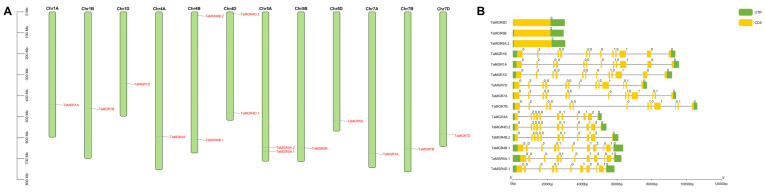
Analysis of chromosomal localization and gene structures. (**A**) Chromosomal localization of *TaMGRs*. The green lines represent chromosomes, and the red fonts indicate *TaMGRs* members. (**B**) Analysis of gene structures of *TaMGRs* members.

The chromosomal locations of wheat *MGR* family members were predicted using TBtools software based on the wheat genome GFF3 file. A total of 15 genes were mapped to different positions on 12 wheat chromosomes (Figure 4A). As a hexaploid species, the *TaMGRs* of wheat were concentrated on chromosomes of 1, 4, 5 and 7 groups (Chr1A–Chr1D, Chr4A–Chr4D, Chr5A–Chr5D and Chr7A–Chr7D). Specifically, chromosomes 4B, 4D and 5A each contained two genes, and the remaining chromosomes each had one gene, indicating an uneven distribution. Thus, chromosomes 4B, 4D and 5A may be the core regions where *TaMGRs* exert their functions. Additionally, there were five genes distributed on each of these four subgenomes. This study analyzed the gene structure of the *TaMGR* family. The results are shown in Figure 4B. Different members exhibited significant differences as well as conservation in gene structure, which was reflected in the structural differences among different subgroups and the consistency in intron phases among subgroup members. The distribution characteristics of untranslated regions (UTRs) and CDSs reflect the functional differentiation within the family and the stability of the core functions of homologous genes. Members of the same subfamily shared similar gene structure characteristics. For example, the CDSs and UTRs of *TaMGR5D*, *TaMGR5B* and *TaMGR5A.2* in Subgroup I were continuously distributed, with relatively simple exon–intron compositions. However, most members, such as *TaMGR1B* and *TaMGR1A*, exhibited complex fragmented exon distribution patterns, where CDS fragments were separated by multiple introns. Except for *TaMGR5D* in Subgroup I, which only had one UTR, all other members and members of other subgroups had two UTRs. All members of Subgroup III contained 10 introns and 11 exons.

### 3.4. Gene Duplication Events and Synteny Analysis

**Figure 5:**
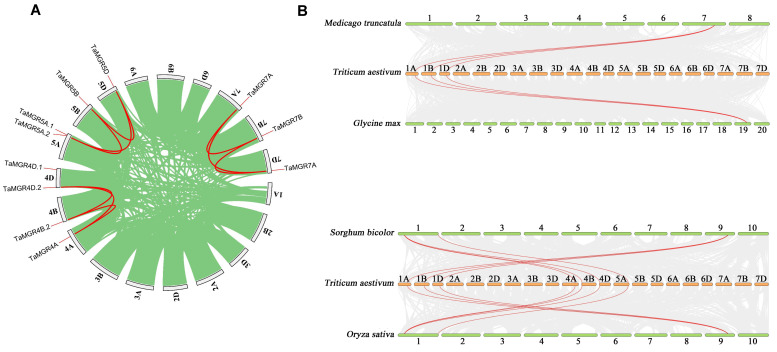
Gene duplication events in the *TaMGR* gene family (**A**). Collinearity analysis of the *TaMGR* gene family between monocots and dicots (**B**).

As a key mechanism, gene duplication leads to new genes, which may possess similar or distinct functions, thereby enabling gene family expansion [51]. Gene duplication can be classified into tandem and segmental duplication [52]. In this study, the MCScanX tool in TBtools software was used to analyze gene duplication events occurring in the *TaMGR* gene family. A total of nine duplicated gene pairs were detected, including one tandem duplication, and the remaining pairs were associated with segmental duplication events (Figure 5A). To clarify the evolutionary relationships of *TaMGRs*, collinearity analyses were performed based on genomic data, including collinearity analyses of wheat with *Sorghum bicolor*, *Oryza sativa*, *Medicago truncatula* and *Glycine max* (Figure 5B). There were three homologous *MGR* gene pairs between *TaMGRs* and the dicotyledonous plants (*Medicago truncatula* and *Glycine max*) and seven homologous *MGR* gene pairs between *TaMGRs* and monocotyledonous plants (*Sorghum bicolor* and *Oryza sativa*). The number of homologous *MGR* gene pairs between *TaMGRs* and monocots was higher than that between *TaMGRs* and dicots. This indicates that the *MGR* family may be highly conserved in monocotyledonous plants, whereas gene copy loss or accelerated sequence divergence may have occurred in dicotyledonous plants.

### 3.5. Analysis of Cis-Acting Elements

**Figure 6:**
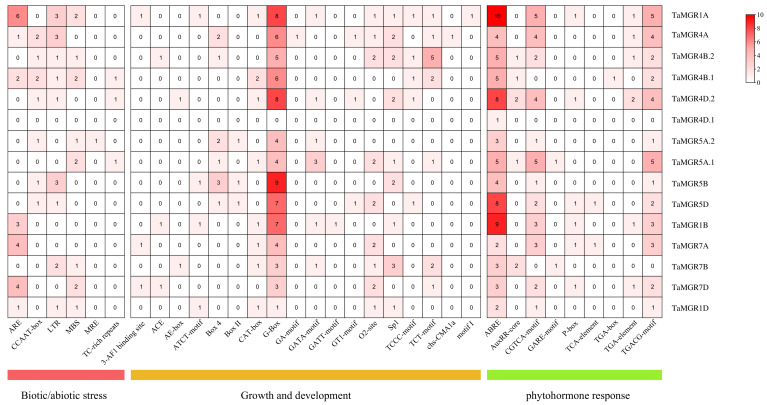
Analysis of cis-acting elements of *TaMGR* genes.

Cis-acting elements in the promoter region play a crucial role in deciphering gene regulatory mechanisms under natural conditions. The potential cis-acting elements were identified by analyzing the 2000bp DNA sequences upstream of 15 genes in the wheat *MGR* family. In this study, the cis-acting elements in the *TaMGRs* promoter regions were classified into three subcategories: biotic and abiotic stress-responsive, growth and development, and phytohormone response elements (Figure 6). Both the total number and classification of cis-acting elements varied among *TaMGR* family members. At the stress response level, ARE, LTR and MBS elements were enriched. *TaMGR1A*, *TaMGR1B*, *TaMGR7A* and *TaMGR7D* showed a relatively high enrichment of AREs. As anaerobic-responsive elements, AREs may be involved in the regulation of gene expression under hypoxic conditions. The presence of LTRs and MBSs indicates that the corresponding genes are involved in the regulation of genes under low-temperature and drought conditions, respectively [53,54,55]. Among the growth and development elements, the high frequency of CAT-box, G-box, O2-site, SPI and other elements in *TaMGRs* indicates their crucial roles in seed development processes. The presence of CAT-box indicates that *TaMGR* genes actively participate in the regulation of meristem-related gene expression [56]. As a crucial light-responsive element during growth and development stages, the G-box accounted for more than 90% of the growth and development-related elements. Among the 15 *TaMGR* members, only one did not contain this element. In terms of phytohormone response, the ABRE achieved 100% coverage among *TaMGRs*, with high enrichment in individual members. For instance, there were 10 ABREs in *TaMGR1A*, 9 in *TaMGR1B*, and 8 each in *TaMGR4D.2* and *TaMGR5D.* The GARE-motif and TGACG-motif were present in most TaMGR members and may be associated with gibberellin signal transduction and jasmonic acid-mediated signaling pathways, respectively [57,58].

### 3.6. Analysis of TaMGRs Expression Patterns

**Figure 7:**
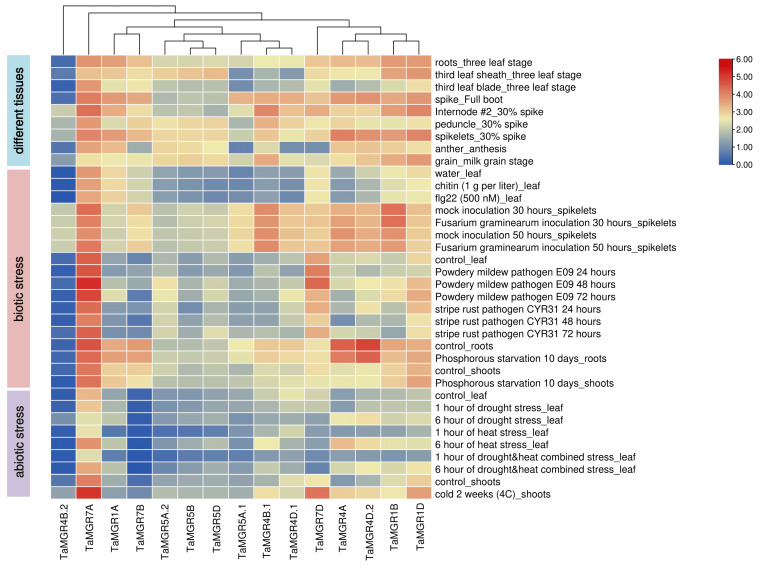
Gene expression analysis of the wheat *MGR* family in different tissues and under different stress treatments.

To investigate the gene expression patterns of the wheat MGR family across different tissues and under various stress treatments, we generated an expression heatmap based on RNA-seq data. The heatmap was organized into three modules, representing the expression profiles of *TaMGR* genes in different tissues, as well as under biotic and abiotic stresses (Figure 7). The results revealed differential expression of *TaMGR* family members in roots, leaves, sheaths, spikes and seeds. Among these, *TaMGR1B*, *TaMGR1D* and *TaMGR7A* exhibited high expression levels across all tissues. *TaMGR4B.1*, *TaMGR4B.2*, and *TaMGR7D* were primarily expressed in the internode, with very low or no expression in other tissues, suggesting potential roles in internode development. *TaMGR5A.1*, *TaMGR4D.1* and *TaMGR7B* were mainly expressed in the spike, with minimal or no expression elsewhere, indicating possible involvement in spike development. *TaMGR4A* was predominantly expressed in spikelets and showed lower expression in other tissues, implying a potential role in spikelet development. Collectively, these findings suggest that the differential expression of *TaMGR* family genes may play important and distinct roles in the growth and development of different wheat tissues.

Furthermore, under treatments with chitin, flg22 and *Fusarium graminearum*, no significant induction of any *TaMGR* family genes was observed. In response to powdery mildew infection, *TaMGR1A*, *TaMGR5A.2*, *TaMGR5D*, *TaMGR7D*, *TaMGR4D.2*, *TaMGR1B* and *TaMGR1D* showed an upward trend in expression in leaves. Following stripe rust infection, *TaMGR5D* and *TaMGR5A.2* exhibited increased expression levels, which gradually elevated with the progression of infection time. These results suggest that *TaMGR5D* and *TaMGR5A.2* may play important roles in wheat’s response to powdery mildew and stripe rust stresses.

Next, we analyzed the expression patterns of *TaMGR* family genes under abiotic stress conditions. Under phosphorus deficiency, no significant induction of *TaMGR* genes was observed overall; however, *TaMGR5A.2* in roots and *TaMGR4D.1* and *TaMGR5A.1* in shoots showed an upward expression trend, suggesting their potential involvement in the wheat phosphorus deficiency response. Under drought stress, *TaMGR4A* and *TaMGR4D.2* were significantly induced in leaves. Under heat stress, *TaMGR7A*, *TaMGR4B.1* and *TaMGR4A* were significantly induced. Notably, under combined drought and heat stress, only *TaMGR4A* was significantly induced, indicating that this gene may play a crucial role in wheat’s response to drought, heat and their combination. Under cold treatment, *TaMGR7A*, *TaMGR4B.1*, *TaMGR7D*, *TaMGR4A*, *TaMGR4D.2* and *TaMGR1D* showed significant upregulation in shoots, implying that these genes may enhance their expression to cope with low-temperature stress.

**Figure 8:**
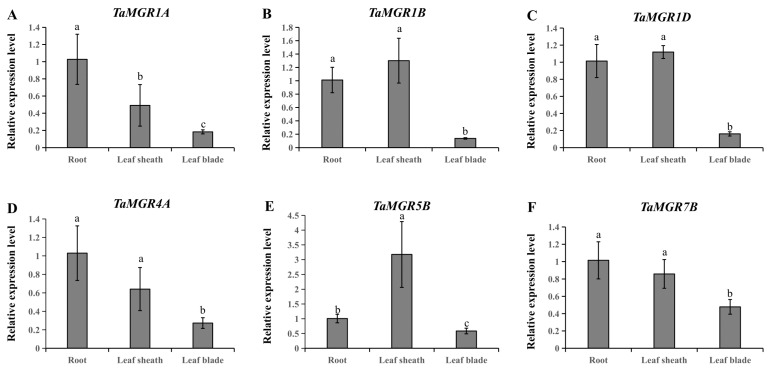
Gene expression analysis of *TaMGRs* in different tissues. (**A**), *TaMGR1A* expression. (**B**), *TaMGR1B* expression. (**C**), *TaMGR1D* expression. (**D**), *TaMGR4A* expression. (**E**), *TaMGR5B* expression. (**F**), *TaMGR7B* expression. The root, leaf sheath and leaf blade of 14-day-old wheat seedlings were used for gene expression analysis. Data represent means ± (SD) of three biological replicates. Different lowercase letters indicate significant differences (*p* < 0.05).

To validate the transcriptome data, we randomly selected six genes (*TaMGT1A*, *TaMGT1B*, *TaMGT1D*, *TaMGT4A*, *TaMGT5B* and *TaMGT7B*) from the *TaMGR* family and analyzed their expression in root, leaf sheath, and leaf tissues (Figure 8). The results demonstrated that the expression trends of these genes across different tissues were consistent with the transcriptome analysis.

## 4. Discussion

Mg transporters are key proteins in plants that maintain Mg homeostasis. The CorA/MGT/MRS2 family has been relatively well-studied, and its members have been identified in *Arabidopsis thaliana* [14], rice [59], maize [22], and soybean [26]. However, research on the MGR family remains limited to *Arabidopsis thaliana* [27], and the member composition, evolutionary characteristics, and functions of this family in wheat have not been reported until now. As a globally important hexaploid food crop, the Mg nutrition status of wheat affects its photosynthetic efficiency and yield. Previous studies have only focused on the MGT family [36], making it difficult to comprehensively decipher the molecular mechanism of Mg transport in wheat. The aim of this study was to fill the research gap in the wheat MGR family and provide a theoretical basis for revealing the regulatory network of Mg transport in wheat.

This study identified 15 *TaMGR* genes in wheat using bioinformatics methods and systematically analyzed their gene structures, chromosomal locations, evolutionary relationships, and other characteristics. TaMGR family members contained 3 or 4 transmembrane domains, with each transmembrane domain spanning 22 amino acids (Figure 1). This observation was consistent with the characteristics previously reported for the *Arabidopsis thaliana* MGR family [27]. However, the CBS domain of MGR at the C-terminus in the *Arabidopsis thaliana* exhibits low conservation, whereas the C-terminal CBS domains in wheat were highly conserved. This interspecific difference in the conservation of the C-terminus may reflect the “fine-tuning” of Mg transport regulatory mechanisms by different species to adapt to their unique physiological environments [60]. TaMGRs were predicted to localize to the plasma membrane, vacuolar membrane, and chloroplasts (Supplementary Table S1), which is consistent with the previously reported subcellular localization of the *Arabidopsis thaliana* MGR family [27]. Phylogenetic analysis revealed that the wheat MGR family comprises 15 members (Figure 2), a number significantly higher than that in *Arabidopsis thaliana*, *Oryza sativa*, *Medicago truncatula, Sorghum bicolor* and *Solanum lycopersicum*, which may be a result of wheat’s polyploidization [61]. This is consistent with the previously reported evolutionary pattern of the NTR gene family (another transporter family in wheat) [62]. The results of motif and gene structure analyses in Figure 3 showed that the absence of motif16 and motif17 in *TaMGR7A*, *TaMGR7B*, and *TaMGR7D* and the absence of motif9 in *TaMGR4D.1* may be the result of genetic recombination, and their outcomes require further experimental verification [63].

The 15 genes identified in wheat exhibited a clustering pattern of individual members on chromosomes 4B, 4D and 5A (Figure 4A). This differs from the clustered distribution of individual members of the TaMGT family on chromosomes 3A, 3B and 3D [36], indicating the evolutionary divergence of these two Mg transporter families in the wheat genome. The high gene structure similarity among members of the same subfamily—such as the continuous distribution of CDSs/UTRs in Subgroup I members and the high conservation of the number of introns in Subgroup III members (Figure 4B)—suggests that these members originated from relatively recent gene duplication events and inherited the basic structural framework of their common ancestor during evolution. This structural conservation, particularly the consistency in exon phases, indicates the stability of the core functions of this subfamily [64]. Most members exhibited a complex pattern of exon fragmentation, which may be a crucial strategy for genes to increase transcript and protein diversity to adapt to complex physiological demands through alternative splicing events [65]. All members of this family, except *TaMGR5D*, possessed both 5′ and 3′ UTRs, indicating that the expression of genes in this family is influenced by post-transcriptional regulatory mechanisms [66]. Among the duplicated gene pairs identified in this study, only one was a tandem duplication, and all others were segmental duplications (Figure 5). The number of segmental duplications far exceeded that of tandem duplications, suggesting that segmental duplication may be the main mechanism for the expansion of the *TaMGRs* in wheat [51]. The transcriptional regulation of gene expression is largely determined by promoters; therefore, conducting research on the cis-acting elements of promoters is a key step in understanding gene regulatory mechanisms [67]. In this study, cis-acting elements related to stress response, hormone response and growth and development were predicted in *TaMGRs* gene promoter regions (Figure 6). The wheat *MGR* family exhibited a high responsiveness to these elements. For instance, the G-box element—a light-responsive element during growth and development—was found in more than 90% of family members, indicating that the expression regulation of the *TaMGR* family is closely related to light [68]. ABREs, a key cis-acting element in the abscisic acid (ABA) signaling pathway, were present in all *TaMGRs*. ABREs can be specifically recognized and bound by AREBs/ABFs, the core transcription factors of the ABA signaling pathway [69]. This pattern of conservation among family members strongly suggests that the *TaMGR* family may be a cluster of common target genes in the ABA signaling pathway [70], functioning when plants respond to physiological demands or environmental stressors. However, the prediction results of these regulatory elements require experimental verification.

Mg transporters are involved in plant responses to adverse stress conditions [15,20]. Transcriptome analysis revealed significant differences in the expression of wheat *TaMGRs* gene family under various environmental stresses (Figure 7). Regarding biotic stresses, powdery mildew or stripe rust infection significantly induced the expression of *TaMGR5D* and *TaMGR5A.2*, suggesting that they may play important roles in wheat’s defense against these plant pathogens, though the specific molecular mechanisms require further investigation. Currently, there are few reports on the involvement of Mg transporters in biotic stress responses. Studies have shown that the rice chloroplast Mg transporter gene *OsMGT3* regulates broad-spectrum disease resistance by influencing salicylic acid accumulation [71]. In terms of abiotic stresses, existing reports and studies have mainly focused on the responses of *MGT* family genes to salt, aluminum toxicity and Mg deficiency [20,72,73], while reports on other stress conditions remain scarce. Differential transcriptome analysis indicated that drought treatment induced the expression of *TaMGR4A* and *TaMGR4D.2* in leaves; heat stress induced the expression of *TaMGR7A*, *TaMGR4B.1*, and *TaMGR4A*; and combined drought and heat treatment induced *TaMGR4A* expression. The molecular mechanisms by which these genes participate in abiotic stress responses await further exploration.

## 5. Conclusions

In this study, a systematic phylogenetic analysis was performed on 15 *TaMGR* members. We clarified the chromosomal localization characteristics of these family members and revealed that the promoter regions were enriched with many cis-acting elements related to stress and hormone responses, providing a theoretical basis for further research on this family in wheat. Transcriptome analysis revealed that *TaMGRs* display distinct expression patterns and significant responsiveness across different tissues at various developmental stages, as well as under diverse stress conditions in wheat. However, this study did not conduct in vitro experiments or in vivo functional verification to clarify the functional mechanisms of *TaMGRs* in wheat Mg transport and stress response. Therefore, the biological functions of the *TaMGRs* should be further investigated.

## Data Availability

The original contributions presented in this study are included in the article/Supplementary Materials. Further inquiries can be directed to the corresponding authors.

## Supplementary Materials

The following supporting information can be downloaded at: https://www.mdpi.com/article/10.3390/cimb47110882/s1.
