# Supplementary material for: Genome-Wide Identification, Phylogeny and Expression Analysis of the Magnesium Release Gene Family in Wheat (Triticum aestivum L.)
Source: Curr Issues Mol Biol. 2025 Oct 23;47(11):882. doi: 10.3390/cimb47110882 (PMC12651028; doi:10.3390/cimb47110882)
Supplement: Supplementary file 1 [file cimb-47-00882-s001.zip › Supplementary Table S1.pdf]

Supplementary Table S1. Predicted information of the wheat MGR family members.

Gene name	Gene ID	Chr	Length	MW	TM	DM	Pl	I.I	GRAVY	Sub
TaMGR1A	TraesCS1A03G0642300.1	Chr1A:441852365-441861925	565	60310.37	3	CBS	6.49	43.34	0.106	VM、PM
TaMGR1B	TraesCS1B03G0728600.1	Chr1B:461105734-461115085	565	60268.41	3	CBS	6.49	43.68	0.125	VM、PM
TaMGR1D	TraesCS1D03G0599200.1	Chr1D:342526171-342535337	565	60321.41	3	CBS	6.48	43.37	0.104	VM、PM
TaMGR4A	TraesCS4A03G0742500.1	Chr4A:593484357-593489468	420	46344.92	3	CBS	5.25	41.54	0.124	VM、PM
TaMGR4B.1	TraesCS4B03G0827330.1	Chr4B:608283803-608290254	510	55623.49	3	CBS	7.71	54.85	-0.048	VM、PM
TaMGR4B.2	TraesCS4B03G0046500.1	Chr4B:17820034-17826104	420	46362.12	3	CBS	5.33	43.36	0.140	VM、PM
TaMGR4D.1	TraesCS4D03G0737800.1	Chr4D:481183869-481189867	510	55525.34	3	CBS	8.13	52.81	-0.057	VM、PM
TaMGR4D.2	TraesCS4D03G0031700.1	Chr4D:8785782-8791168	420	46232.88	3	CBS	5.33	42.88	0.149	VM、PM
TaMGR5A.1	TraesCS5A03G1157000.1	Chr5A:663966534-663972831	509	55323.07	3	CBS	8.13	52.93	-0.046	VM、PM
TaMGR5A.2	TraesCS5A03G1094700.1	Chr5A:645355114-645358131	723	78186.31	4	CBS	4.77	47.46	-0.024	Chl
TaMGR5B	TraesCS5B03G1160900.1	Chr5B:648349236-648352161	696	75541.28	4	CBS	4.70	44.21	-0.011	Chl
TaMGR5D	TraesCS5D03G1052700.1	Chr5D:518697059-518700049	723	78538.71	4	CBS	4.75	46.78	-0.043	Chl
TaMGR7A	TraesCS7A03G1158400.1	Chr7A:676059782-676069187	518	55344.11	3	CBS	5.92	36.16	0.172	VM、PM
TaMGR7B	TraesCS7B03G1025300.1	Chr7B:652045409-652056029	518	55377.12	3	CBS	5.92	38.13	0.172	VM、PM
TaMGR7D	TraesCS7D03G1106100.1	Chr7D:581989296-581997021	518	55332.05	3	CBS	5.92	36.01	0.162	VM、PM

Chr： chromosome; Length :amino acid length; MW： molecular; weigh; TM: Transmembrane structural domain; DM: domain; PI: Isoelectric point; I.I: Instability index; GRAVY: Grand average of hydropathy; Sub: Subcellular localization; VM: vacuolar membrane; PM: plasma membrane; Chl: chloroplast.
