# Supplementary material for: Genome-Wide Identification, Phylogeny and Expression Analysis of the Magnesium Release Gene Family in Wheat (Triticum aestivum L.)
Source: Curr Issues Mol Biol. 2025 Oct 23;47(11):882. doi: 10.3390/cimb47110882 (PMC12651028; doi:10.3390/cimb47110882)
Supplement: Supplementary file 1 [file cimb-47-00882-s001.zip › Supplementary Table S3.pdf]

Supplementary Table S3. PCR primers used for Real-Time PCR

Gene name	Forward (5'-3')	Reverse (5'-3')
TaMGT1A	AATGTTATTGGCCTCCTGTT	ACTCCATTCGATGGCTTATC
TaMGT1B	TGCTTTTGGGGAGGTTATAC	AGTCCAAAGCTCCACTTATG
TaMGT1D	ACATTTGTTCTTGCTTTCGG	AGTCCAAAGCTCCACTTATG
TaMGT4A	GCTAAGATATTGCCCGTCG	ATTTCCATGCAGTGTCACAA
TaMGT5B	CCCTGTTACTCGGTCTCT	AACGGGATACAGGATGATTG
TaMGT7B	TCGTCATGTTCATTGCCTAT	AACATTTCTAGGGCTTCCTG
Ta2291	GCTCTCCAACAACATTGCCAAC	GCTTCTGCCTGTCACATACGC
